# Pre-pregnancy weight status, early pregnancy lipid profile and blood pressure course during pregnancy: The ABCD study

**DOI:** 10.1371/journal.pone.0177554

**Published:** 2017-05-19

**Authors:** Adriëtte J. J. M. Oostvogels, Wim B. Busschers, Eline J. M. Spierings, Tessa J. Roseboom, Maaike G. J. Gademan, Tanja G. M. Vrijkotte

**Affiliations:** 1 Department of Public Health, Academic Medical Centre, University of Amsterdam, Amsterdam, The Netherlands; 2 Department of Gynaecology and Obstetrics, Academic Medical Centre, University of Amsterdam, Amsterdam, The Netherlands; 3 Department of Clinical Epidemiology, Biostatistics and Bioinformatics, Academic Medical Centre, University of Amsterdam, Amsterdam, The Netherlands; University of North Carolina at Chapel Hill, UNITED STATES

## Abstract

**Objective:**

Although pre-pregnancy weight status and early pregnancy lipid profile are known to influence blood pressure course during pregnancy, little is known about how these two factors interact. The association between pre-pregnancy weight status and blood pressure course during pregnancy was assessed in the prospective ABCD study and the role (independent/mediating/moderating) of early pregnancy lipid profile in this association was determined.

**Methods:**

We included 2500 normal weight (<25 kg/m2) and 600 overweight (≥25 kg/m2) women from the prospective ABCD-study with available measurements of non-fasting early pregnancy lipids [median (IQR): 13 (12–14) weeks of gestation] and blood pressure during pregnancy [mean (SD) = 10 (2.3)]. Lipids (triglycerides, total cholesterol, apolipoprotein A1, apolipoprotein B and free fatty acids) were divided into tertiles. Multilevel piecewise linear spline models were used to describe the course of systolic and diastolic blood pressure (SBP/DBP) in four time periods during gestation for overweight and normal weight women.

**Results:**

Both SBP (5.3 mmHg) and DBP (3.9 mmHg) were higher in overweight compared to normal weight women and this difference remained the same over all four time periods. The difference in SBP and DBP was not mediated or moderated by the lipid profile. Lipid profile had an independent positive effect on both SBP (range 1.3–2.2 mmHg) and DBP (0.8–1.1 mmHg), but did not change blood pressure course.

**Conclusions:**

Both pre-pregnancy weight status and early pregnancy lipid profile independently increase blood pressure during pregnancy. Improving pre-pregnancy weight status and early pregnancy lipid profile might result in a healthier blood pressure course during pregnancy.

## Introduction

Overweight or obesity before pregnancy is associated with hypertensive disorders of pregnancy,[1, 2] which in turn are associated with maternal, foetal and infant mortality.[3, 4] Therefore, identifying modifiable risk factors affecting the blood pressure (BP) course in pregnancy is important as they may be instrumental in devising new preventive and therapeutic strategies.

Overweight and obese women display a different BP course than normal weight women.[5–7] Normally, blood pressure decreases from the start of pregnancy under the influence of progesterone and prostaglandins, and due to increased uteroplacental circulation,[8] reaching a nadir in midpregnancy.[9] From here BP increases to normal pre-pregnancy blood pressure at term [9] as blood volume increases to compensate for the increased uteroplacental circulation and for blood loss at delivery.[8] Compared to normal weight women, overweight and obese women start their pregnancy with a higher BP, have a smaller BP decrease to midpregnancy and a faster increase until the end of pregnancy.[5, 6]

Overweight and obese women also have a more atherogenic lipid profile throughout pregnancy.[10–12] A more atherogenic lipid profile in pregnancy is associated with hypertensive disorders of pregnancy, independent of maternal weight status.[13–17] The lipid profile changes during pregnancy: in early pregnancy lipids are low and an accumulation of maternal fat depots is followed by increased adipose tissue lipolysis and subsequent hyperlipidaemia in late pregnancy.[11, 12] At this stage, triglyceride-rich particles and related remnants can damage the endothelium through lipid-mediated oxidative stress mechanisms,[18, 19] leading to higher BP.[20] Although associations between a more atherogenic lipid profile and hypertensive disorders of pregnancy have been reported, the association between early pregnancy lipid profile and BP course, especially in the first half of pregnancy, remains unknown.

To our knowledge, the role of both pre-pregnancy weight status and early pregnancy lipid profile on BP course during pregnancy has not yet been studied. Therefore, this study aimed to describe the association between pre-pregnancy weight status and BP course and the role of early pregnancy lipid profile: i.e. does the lipid profile mediate or moderate this association? The present study used data from the large Dutch prospective ABCD study to examine these associations. Based on the literature, we hypothesise that overweight women, partly due to their more atherogenic lipid profile, start their pregnancy with a higher BP and have a smaller decrease in BP in the first half of the pregnancy, but a greater increase in the second half of the pregnancy (mediation). Moreover, we expect to find the most detrimental outcomes (i.e. the highest absolute BP with the steepest increase in BP during pregnancy) in overweight women with a more atherogenic lipid profile (moderation).

## Methods

### The ABCD study

This study was embedded in the prospective Amsterdam Born Children and their Development (ABCD) study (http://www.abcd-study.nl). Details on the study design are described elsewhere.[21] This study was conducted according to the guidelines of the Declaration of Helsinki and all procedures were approved by the Central Committee on Research Involving Human Subjects, the Medical Research Ethics Committees of the participating hospitals, and the Registration Committee of the municipality of Amsterdam.

### Study population

Between January 2003 and March 2004 all pregnant women living in Amsterdam were invited to participate in the ABCD study at their first antenatal screening (median: 13 (IQR: 12–14) weeks of gestation). All women were asked to participate in the ABCD biomarker study and to fill out a pregnancy questionnaire, which contained an informed consent to grant permission for perusal of their medical files. Of all 12,373 pregnant women approached, 8266 returned the pregnancy questionnaire (response rate 67%) at an average of 16 weeks gestation [inter quartile range (IQR) 14–18 weeks] and 4389 women also participated in the biomarker study (53%). For this study, non-fasting blood sampling (10-ml EDTA and 9-ml serum) took place during routine blood collection for screening purposes [median: 13 (IQR: 12–14) weeks of gestation] of which 4269 had reliable and available measurements of one of the lipids used in the present study. A total of 7043 women (85%) gave permission to collect data from their medical files and, of these, the medical files of 6741 women (96%) could be traced.

**Fig 1:**
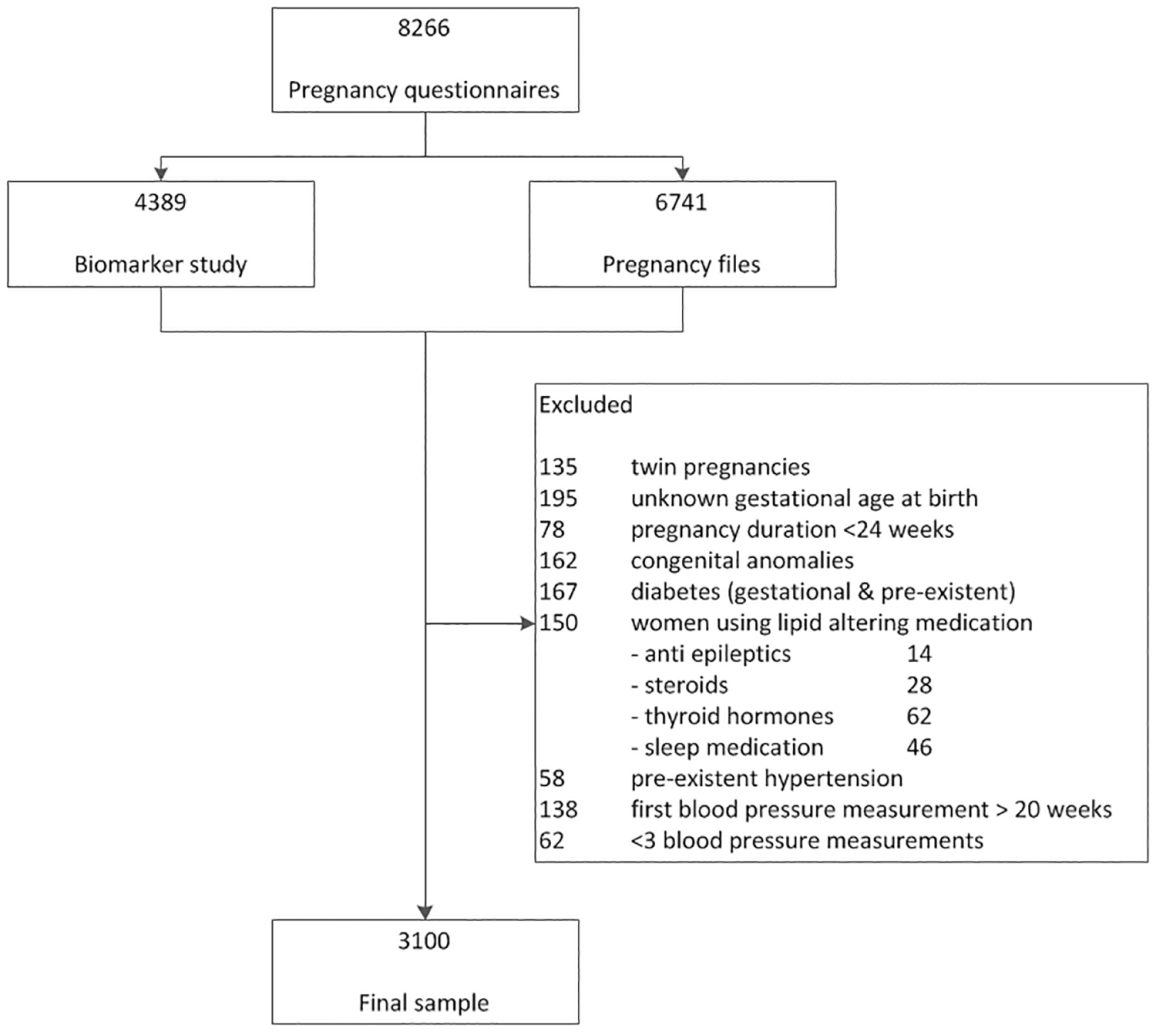
Flowchart of inclusion of the study participants.

Excluded from the study were women carrying twins or who had no data on the gestational age at blood sampling, women with diabetes (both pre-existent and pregnancy-induced), with pre-existing hypertension, and those using lipid-altering medication (e.g. antiepileptic drugs, antidepressant, steroids, thyroid hormones, or sleep medication). Also excluded were women with <3 BP measurements, or with first BP measurement after 20 weeks of gestation. The final sample included 3100 women (Fig 1).

### Pre-pregnancy weight status

Pre-pregnancy body mass index (pBMI) was based on height and weight as reported in the pregnancy questionnaire, completed with data from the pregnancy files. Two categories were formed based on the WHO guidelines: normal weight (<25 kg/m^2^) and overweight (≥25 kg/m^2^).[22]

### Early pregnancy lipid profile

The non-fasting early pregnancy lipid profile consisted of triglycerides (TG), total cholesterol (TC), free fatty acids (FFA), apolipoprotein A1 (ApoA1) and apolipoprotein B (ApoB) and were derived from biochemical analysis. The method of lipid analyses is described in detail elsewhere.[10] Since lipid concentrations increase in the first half of pregnancy, lipid measurements were interpolated to 10 weeks of gestation using a linear regression model.[12]

### BP course during pregnancy

BP was measured at each prenatal visit by the obstetric caregiver (midwife/obstetrician). Blood pressure measurements were done in a seated position using the appropriate cuff size, mostly measured on the right arm. More than 85% of the obstetric caregivers used an auscultatory device to measure blood pressure (using Korotkoff phase I for systolic BP (SBP) and Korotkoff phase V for diastolic BP (DBP)), while the remainder of the obstetrics caregivers used an automated BP monitor. BP and date of measurement were retrieved from obstetric medical files.

A mean of 10 (SD = 2.3) BP measurements per pregnant woman were obtained. On average the first measurement was at 12.9 (SD = 2.0) weeks of gestation and the last at 38 (SD = 3.4) weeks. When medical files stated the use of anti-hypertensive drugs (n = 25) from a certain date onwards [mean gestational age (SD) = 33.6 (3.6) weeks], BP measurements after that date were excluded. Double entry was performed in a randomly selected sample of 315 (5%) records. Agreement on overall BP was high for SBP (97.8%) and DBP (97.2%).

### Covariates

Covariates were chosen a priori based on their association with maternal weight status or BP.[5, 7] Chosen covariates included maternal age (years; continuous), education (years after primary school; continuous); parity (nulliparous/multiparous), ethnicity [Dutch, Surinamese-Hindu, Caribbean (Surinamese-Creole/ Antillean/Aruban), Turkish, Moroccan, Ghanaian, other], and smoking and alcohol use during pregnancy (yes/no) which were self-reported in the pregnancy questionnaire. Ethnicity was based on the country of birth of the pregnant woman’s mother. Gestational age at blood sampling, gestational age at giving birth (days) and foetal sex (male/female) were obtained from the Youth Health Care Registration. Gestational age was based on ultrasound or, when not available (±10%), was calculated by the obstetric care provider, based on the first day of the last menstrual period. Gestational age at blood sampling was calculated based on gestational age at birth, date of birth and date of blood sampling.

### Statistical analysis

Descriptive statistics of the study population, comparing normal weight and overweight women, and the non-response analysis, comparing women in this study with women without either an early pregnancy lipid profile or BP course during pregnancy, were performed using independent samples t-tests for continuous variables and χ2-tests for categorical variables. To reclaim the cases with missing values, multivariate imputation by chained equation (MICE) was used after descriptive analyses. Missings for maternal education (0.61%), ethnicity (0.16%), alcohol use (0.03%) and smoking (0.06%) during pregnancy, foetal sex (0.03%) and pBMI (6.13%) were imputed. [23]

BP course during pregnancy was investigated using a model described by Macdonald-Wallis [7]: as a sequence of individual BP data are likely to be dependent, two-level mixed models were used to account for the possible correlation between the observations within the same woman. Moreover, the model accounted for gestational age at birth, as women with higher BP tend to give birth earlier than women with lower BP.[7, 9] Piecewise linear splines with BP as a function of time with three internal knots, based on the best fitting model, and also allowing for individual departure from the mean slopes [and intercept] were fitted on the study sample of 3100 women. Based on the Akaike's Information Criterion (AIC) the best fitting model with three internal knots for SBP course yielded internal knots at 16.5, 31.5 and 35.5 weeks of gestation, while the best fitting model for DBP was with internal knots at 19.5, 31.5 and 35.5 weeks of gestation.

Associations between weight status, early pregnancy lipid profile and BP course in the four time periods were tested in three subsequent models. First unadjusted associations were tested in a crude model, then adjustments were made for age, education, parity, ethnicity, smoking and alcohol use during pregnancy and foetal sex. To test for differences between normal weight and overweight women in BP course we also fitted a model with interaction between weight status and time period, and compared the model with and without the interaction term through a likelihood ratio test. Finally, we added the early pregnancy lipids, divided into tertiles, separately. Tertiles were chosen as it is difficult to make comparisons in these analyses using the continuous measurements. Moreover, because preliminary analyses revealed little differences in results between quintiles and tertiles, to increase the statistical strength we chose tertiles for further analyses. To test for differences in BP course for women in the different early pregnancy lipid tertiles, we also fitted a model with interaction between tertiles of early pregnancy and time period, and compared the model with and without the interaction term through a likelihood ratio test. Furthermore, to investigate the possible modification by weight status of the above-mentioned association, three-way interaction terms between weight status, lipids and time periods were incorporated into the model. The overall significance of these possible alterations was again assessed using likelihood ratio tests. Sensitivity analyses were performed by repeating all analyses in two subsamples. These subsamples were created by dividing our study population into a subsample with no complications (gestational age at birth ≥37 weeks and no PIH/PE) and a subsample with complications (gestational age at birth <37 weeks and/or PIH/PE).

Descriptive analyses were performed with the SPSS package version 21.0 (SPPS Inc., Chicago IL). The statistical package *R* 2.15.3 with MLWIN plug-in was used to model BP and analyse the associations between pre-pregnancy weight status, early pregnancy lipid profile and BP course during pregnancy. Statistical significance was set at p<0.05.

## Results

### Study population

The study population was divided into pre-pregnancy normal weight (<25 kg/m^2^, n = 2500; 80.6%) and overweight (>25 kg/m^2^, n = 600; 19.4%). Overweight women were less educated, more often multiparous, more often of non-Dutch ethnicity and multiparous, drank less alcohol during pregnancy, and had a more atherogenic lipid profile in early pregnancy (Table 1). Of the overweight women, 118 were obese (19.7%). Our subsample was somewhat older, leaner, higher educated, more often of Dutch origin, more often nulliparous and consumed more alcohol during pregnancy than women in the ABCD study who were not included in the present study because no early pregnancy lipid profile or ≤3 valid BP measurements were available (n = 4061; Fig 1 and S1 Table). For each analysis, women were divided into tertiles based on their lipid levels. Cut-off levels for each lipid are presented in Table 2.

**Table 1:** Characteristics of the study population.

	Normal weight (n = 2500) *%/mean (SD)*	Overweight (n = 600) *%/mean (SD)*	*p-value*
Age (years)	31.1 (4.6)	31.1 (4.9)	P = 0.693
Body mass index (kg/m2)	21.4 (4.6)	28.2 (3.4)	P<0.001
Education (years)	9.8 (3.5)	8.0 (4.1)	P<0.001
Ethnicity			P<0.001
- Dutch	65.3	50.3	
- Surinamese-Hindu	1.5	1.3	
- Black Caribbean	3.1	9.2	
- Turkish	3.6	5.7	
- Moroccan	3.7	13.0	
- Ghanaian	0.4	3.5	
- Other	22.4	17.0	
% Nulliparous	60.6	47.5	P<0.001
% Smoking during pregnancy	9.6	8.3	P = 0.390
% Alcohol consumption during pregnancy	29.0	15.3	P<0.001
Gestational age at lipid measurement (days)	92 (17)	93 (17)	P = 0.163
Triglycerides (mmol/L)*	1.53 (0.59)	1.72 (0.64)	P<0.001
Free fatty acids (mmol/L)*	0.31 (0.16)	0.37 (0.20)	P<0.001
Total cholesterol (mmol/L)*	5.38 (0.98)	5.53 (1.01)	P = 0.001
Apolipoprotein A1 (g/L)*	0.017 (0.002)	0.016 (0.002)	P = 0.069
Apolipoprotein B (g/L)*	0.008 (0.002)	0.009 (0.002)	P<0.001
% Males	49.2	48.8	P = 0.892
Gestational age at birth (days)	279 (13)	279 (15)	P = 0.390
% Preterm	4.6	6.0	P = 0.169

* lipids were interpolated to 10 weeks of gestation

**Table 2:** Tertiles of the investigated lipids#.

	n	Lowest tertile		Middle tertile		Highest tertile	
		Median	Range	Median	Range	Median	Range
**TG (mmol/L)**	3035	1.04	0.28–1.25	1.47	1.25–1.72	2.08	1.72–5.77
**TC (mmol/L)**	3036	4.50	0.48–4.92	5.33	4.92–5.76	6.34	5.76–11.9
**ApoA1 (g/L)**	2177	1.43	0.95–1.54	1.64	1.54–1.74	1.88	1.74–2.87
**ApoB (g/L)**	2193	0.63	0.14–0.72	0.81	0.72–0.89	1.01	0.89–1.96
**FFA (mmol/L)**	2079	0.18	0.06–0.23	0.28	0.23–0.35	0.47	0.35–1.27

ApoA1 = Apolipoprotein A1; ApoB = Apolipoprotein B; FFA = Free Fatty Acids; TC = total cholesterol; TG = triglycerides

^#^ lipids were determined in non-fasting blood samples drawn at a median of 13 (IQR = 12–14) weeks of gestation.

### Blood pressure course during pregnancy of normal weight women

**Fig 2:**
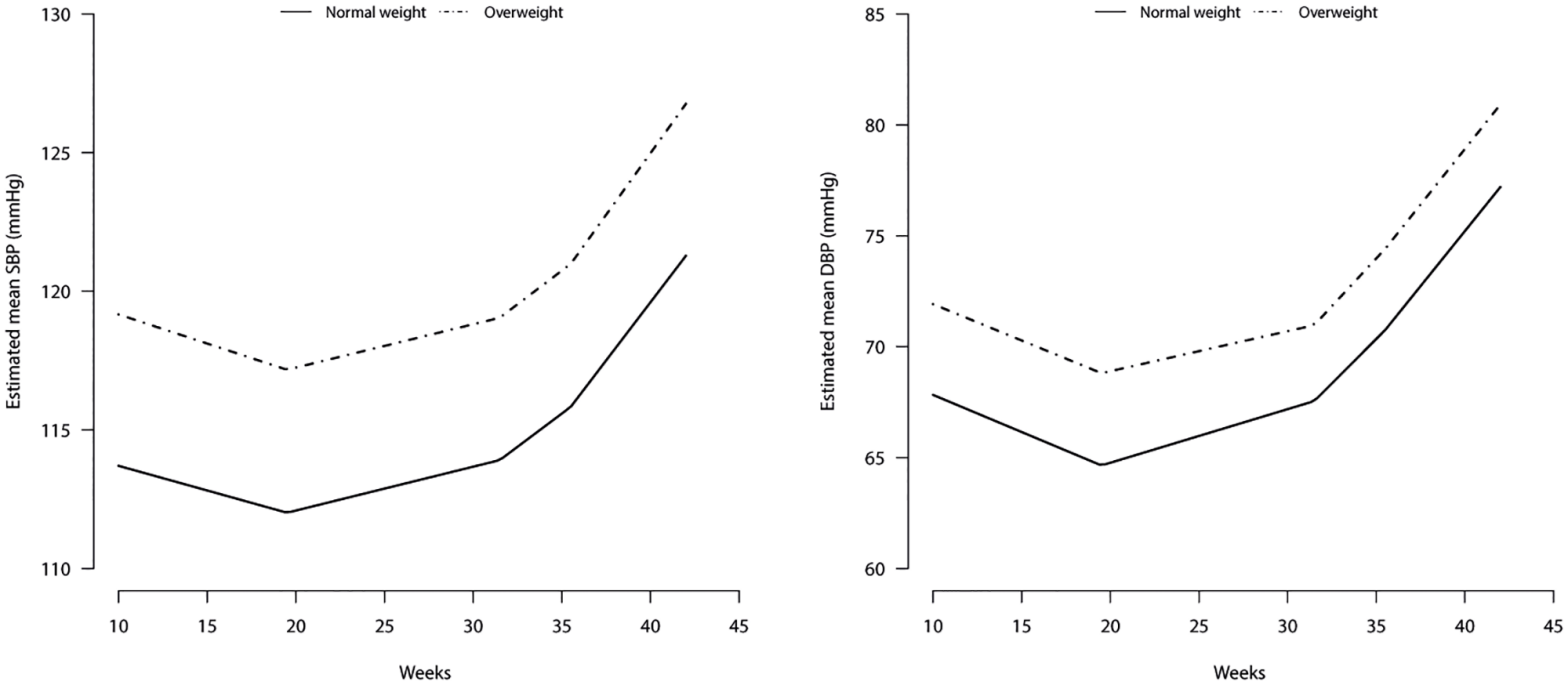
Blood pressure course during pregnancy stratified for women with normal weight and overweight. (A) Systolic blood pressure (SBP): 10–16.5 weeks: estimated β = -0.2 mmHg/week (95% CI: -0.3;-0.1), 16.5–31.5 weeks: estimated β = 0.2 mmHg/week (95% CI: 0.1;0.2), 31.5–35.5 weeks: estimated β = 0.5 mmHg/week (95% CI: 0.4;0.6), 35.5–42 weeks: estimated β = 0.9 mmHg/week (95% CI: 0.7;1.0). (B) Diastolic blood pressure (DBP): 10–19.5 weeks: estimated β = -0.3 mmHg/week (95% CI: -0.4;-0.3), 19.5–31.5 weeks: estimated β = 0.2 mmHg/week (95% CI: 0.2;0.3), 31.5–35.5 weeks: estimated β = 0.8 mmHg/week (95% CI: 0.7;0.9), 35.5–42 weeks: estimated β = 1.0 mmHg/week (95% CI: 0.9;1.1).

For normal weight women their BP course was as follows: starting with an average SBP of 111.3 (SD = 11.1) mmHg and an average DBP of 65.6 (SD = 8.2) mmHg their BP decreased in the first period (10–16.5 weeks) by 0.2 (SBP) and 0.3 (DBP) mmHg/week. During the second (16.5–31.5 weeks), and BP increased by 0.2 (both SBP and DBP) mmHg/week, third (31.5–35.5 weeks) 0.5 (SBP) and 0.8 (DBP) mmHg/week and fourth period (35.5–42 weeks) 0.9 (SBP) and 1.0 (DBP) mmHg/week.(Fig 2).

### Independent effect of pre-pregnancy weight status on BP course during pregnancy

No evidence was found for a difference in BP course between normal weight and overweight women, except for an absolute overall difference, as adding the interaction terms between weight status and time period yielded no significant improvement in the model [p = 0.98 (SBP) and p = 0.30 (DBP)]. During pregnancy, overweight women had on average a 4.3 mmHg higher SBP (95%CI:3.6–5.1) and a 3.3 mmHg higher DBP (95% CI:2.8–3.9). Adjustments for the covariates increased these differences to 5.3 (SBP; 95% CI:4.6–6.0) and 3.9 mmHg (DBP; 95% CI:3.3–4.5) (Fig 2; Tables 3 and 4). Thus, there was no significant difference between the two BP courses, except that overweight women had a higher BP from the start (Fig 2).

**Table 3:** Association between pre-pregnancy BMI and systolic blood pressure course during pregnancy, with maternal lipids.

		Mean difference in average systolic blood pressure (mmHg)				
		pBMI		Lipids#		
		Normal weight	Overweight	Lowest tertile	Middle tertile	Highest tertile
**Model 1**		Ref	4.3 (3.6–5.1)	-	-	-
**Model 2**		Ref	5.3 (4.6–6.0)	-	-	-
**Model 3**	+ TG	Ref	5.2 (4.4–5.9)	Ref	0.6 (-0.1–1.2)	1.7 (1.0–2.4)
	+ TC	Ref	5.2 (4.5–5.9)	Ref	1.2 (0.5–1.9)	1.9 (1.2–2.6)
	+ ApoA1	Ref	5.5 (4.7–6.4)	Ref	0.8 (-0.0–1.6)	1.6 (0.8–2.4)
	+ ApoB	Ref	5.2 (4.3–6.0)	Ref	1.1 (0.3–1.9)	2.2 (1.4–3.0)
	+ FFA	Ref	5.3 (4.4–6.2)	Ref	1.0 (0.2–1.8)	1.3 (0.5–2.2)

Model 1 –crude model; model 2 –adjusted for: maternal age, education, ethnicity, parity, smoking and alcohol use during pregnancy and sex of the foetus; model 3 –adjusted for model 2 + individual lipids separately.

TG = triglycerides (mmol/L); TC = total cholesterol (mmol/L); ApoA1 = Apolipoprotein A1 (mg/dL); ApoB = Apolipoprotein B (mg/dL); FFA = Free Fatty Acids (mmol/L)

^#^ lipids were determined in non-fasting blood samples drawn at a median of 13 (IQR = 12–14) weeks of gestation.

**Table 4:** Association between pre-pregnancy body mass index (pBMI) and diastolic blood pressure course during pregnancy, with maternal lipids.

		Mean difference in average diastolic blood pressure (mmHg)				
		pBMI		Lipids#		
		Normal weight	Overweight	Lowest tertile	Middle tertile	Highest tertile
**Model 1**		Ref	3.3 (2.8–3.9)	-	-	-
**Model 2**		Ref	3.9 (3.3–4.5)	-	-	-
**Model 3**	+ TG	Ref	3.8 (3.3–4.4)	Ref	*	*
	+ TC	Ref	3.9 (3.3–4.5)	Ref	0.3 (-0.2–0.8)	1.1 (0.6–1.6)
	+ ApoA1	Ref	4.1 (3.4–4.7)	Ref	*	*
	+ ApoB	Ref	3.9 (3.2–4.6)	Ref	0.52 (-0.09–1.14)	1.1 (0.4–1.7)
	+ FFA	Ref	4.0 (3.3–4.6)	Ref	*	1.0 (0.3–1.6)

Model 1 –crude model; model 2 –adjusted for: maternal age, education, ethnicity, parity, smoking and alcohol use during pregnancy and sex of the foetus; model 3 –adjusted for model 2 + individual lipids separately.

TG = triglycerides (mmol/L); TC = total cholesterol (mmol/L); ApoA1 = Apolipoprotein A1 (mg/dL); ApoB = Apolipoprotein B (mg/dL); FFA = Free Fatty Acids (mmol/L)

^#^ lipids were determined in non-fasting blood samples drawn at a median of 13 (IQR = 12–14) weeks of gestation.

* Significant interactions between tertiles of lipids and time period; blood pressure course for these tertiles are described in detail in the text

### Mediation of early pregnancy lipid profile in the association between pre-pregnancy weight status and blood pressure course during pregnancy

When early pregnancy lipids were added to the model, the difference in BP during pregnancy between normal weight and overweight women changed only slightly (Tables 3 and 4). Therefore, mediation of maternal lipids in the association between pre-pregnancy weight status and BP course during pregnancy seems unlikely.

### Independent effect of early pregnancy lipid profile on BP course during pregnancy

**Fig 3:**
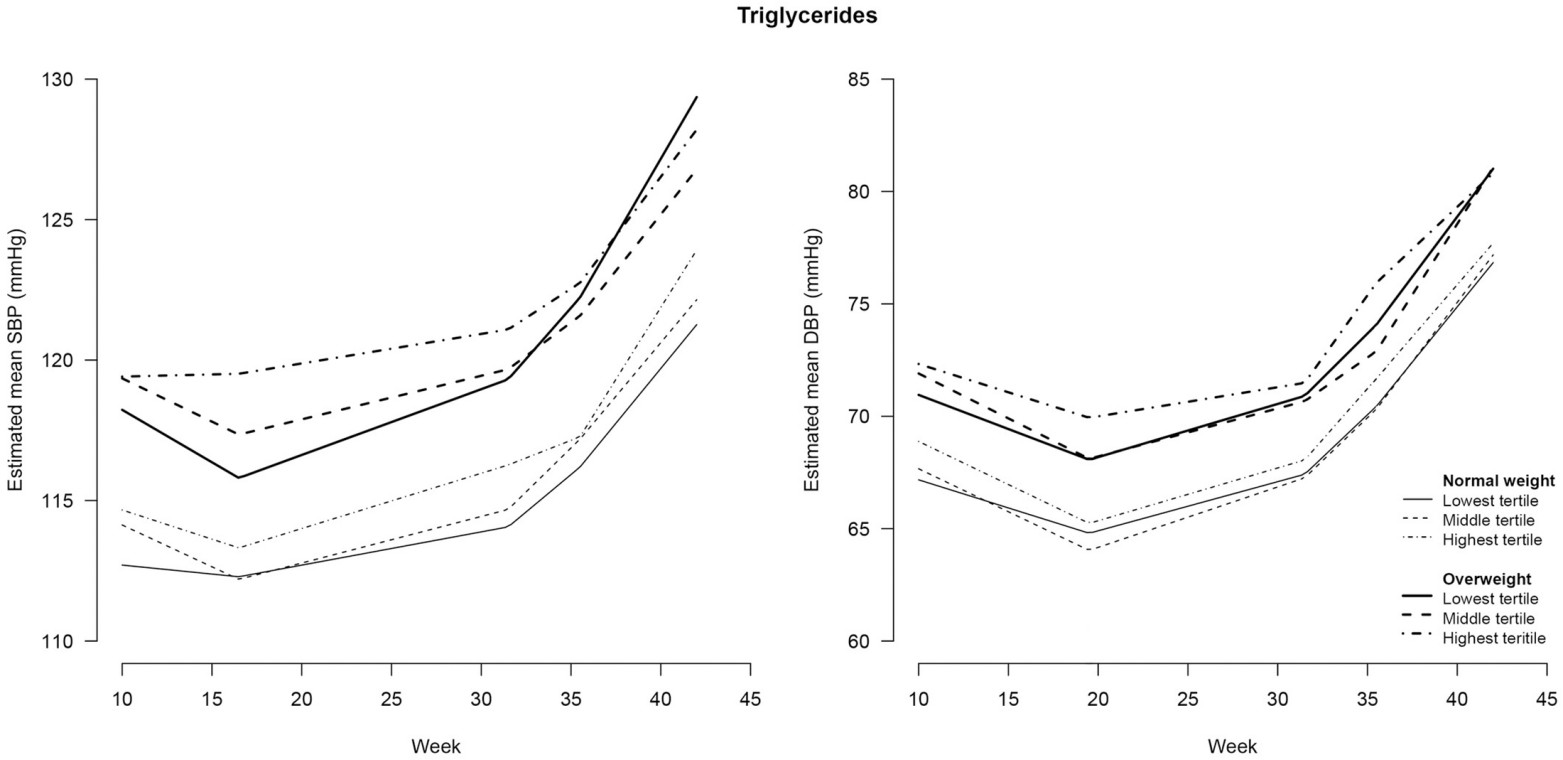
Blood pressure during pregnancy stratified for maternal weight status and tertiles of triglycerides^#^. SBP = systolic blood pressure; DBP = diastolic blood pressure. # triglycerides were determined in non-fasting blood samples drawn at a median of 13 (IQR = 12–14) weeks of gestation.

**Fig 4:**
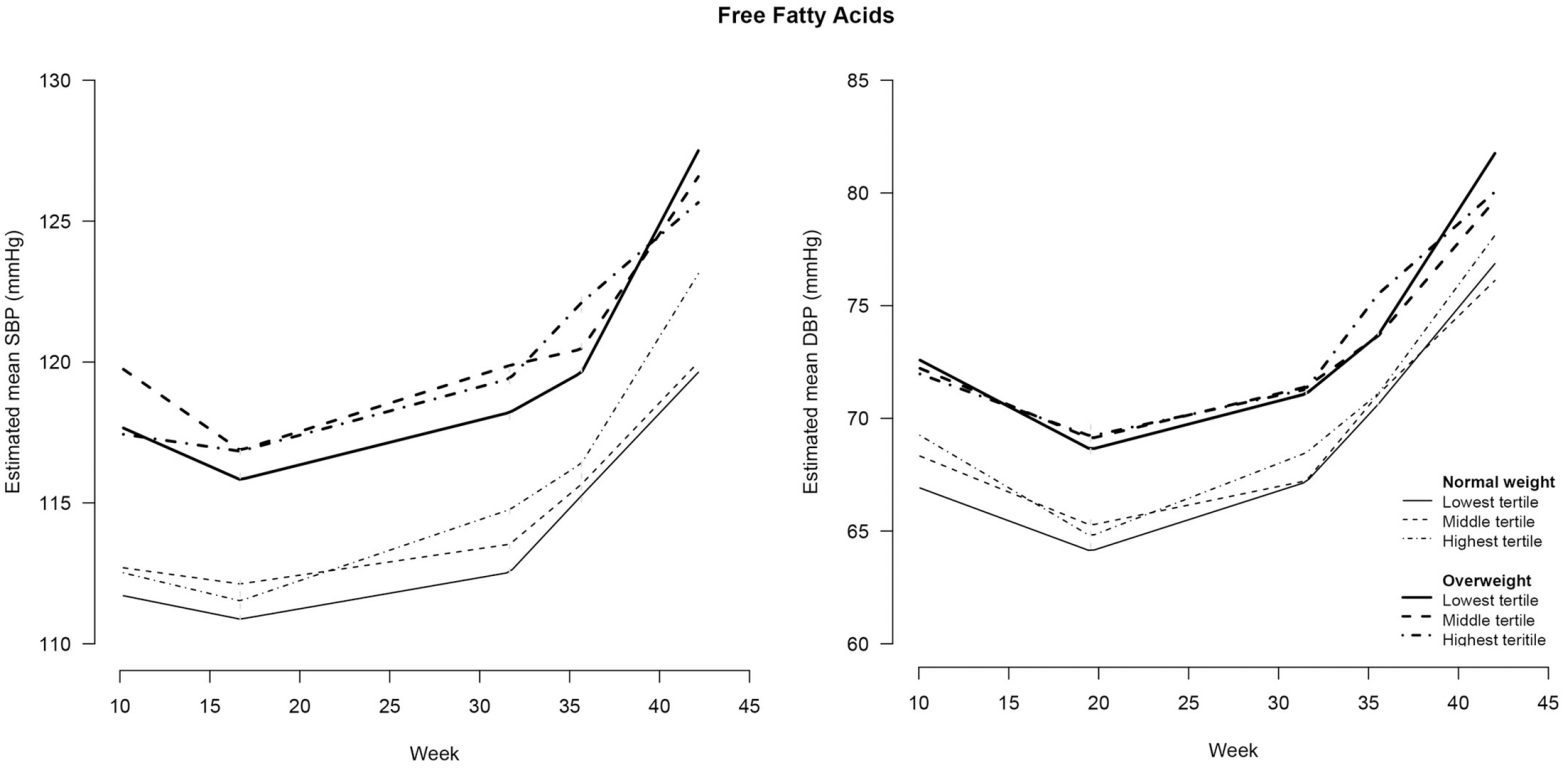
Blood pressure during pregnancy stratified for maternal weight status and tertiles of free fatty acids^#^. SBP = systolic blood pressure; DBP = diastolic blood pressure. # free fatty acids were determined in non-fasting blood samples drawn at a median of 13 (IQR = 12–14) weeks of gestation.

**Fig 5:**
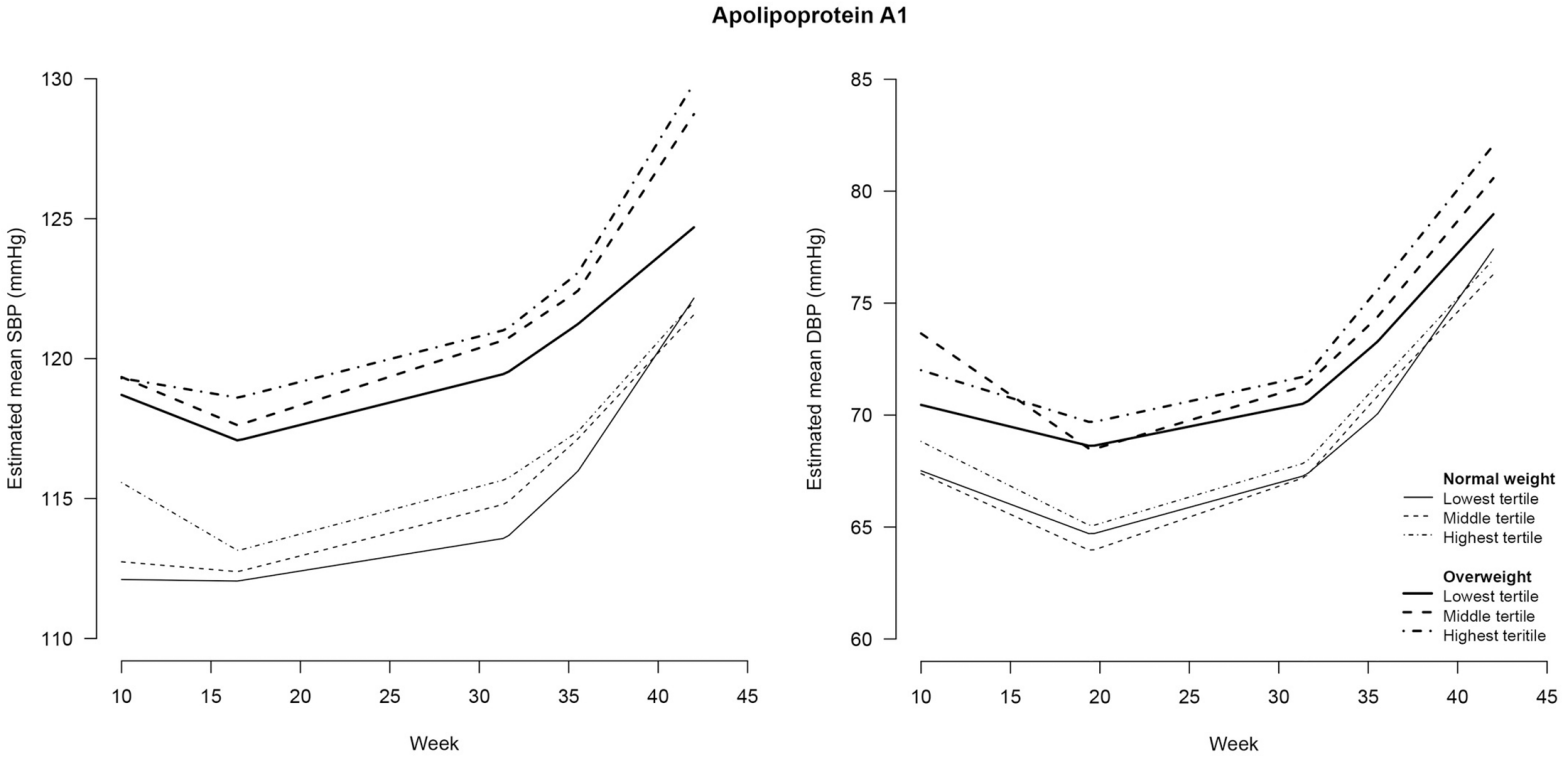
Blood pressure during pregnancy stratified for maternal weight status and tertiles of Apolipoprotein A1^#^. SBP = systolic blood pressure; DBP = diastolic blood pressure. # Apolipoprotein A_1_ was determined in non-fasting blood samples drawn at a median of 13 (IQR = 12–14) weeks of gestation.

Tables 3 and 4 also show the associations between early pregnancy lipids and BP course during pregnancy. During the entire course of pregnancy, all women in the highest lipid tertile had a higher SBP and DBP compared to women in the lowest tertile. Adding interaction terms between lipids and time period did not improve the model for SBP; i.e. women in the different tertiles of the lipids did not show a steeper or less steep increase or decrease in SBP over time. In contrast, the model for DBP was improved when interaction terms between TG and time period (Fig 3), FFA and time period (Fig 4), or ApoA1 and time period (Fig 5) were added to the corresponding model, indicating that women in the different tertiles of these lipids had a different DBP course. For TG, women in the highest tertile had a smaller decrease in the first time period and a steeper increase in the third period than women in the lowest tertile of TG. Women in the highest tertile of ApoA1 had a steeper increase in the third period compared to women in the lowest tertile of ApoA1. Women in the middle tertile of FFA had a smaller increase in DBP in the second time period compared to the women in the lowest tertile of FFA.

### Effect modification of early pregnancy lipid profile on the association between pre-pregnancy weight status and BP course during pregnancy

**Fig 6:**
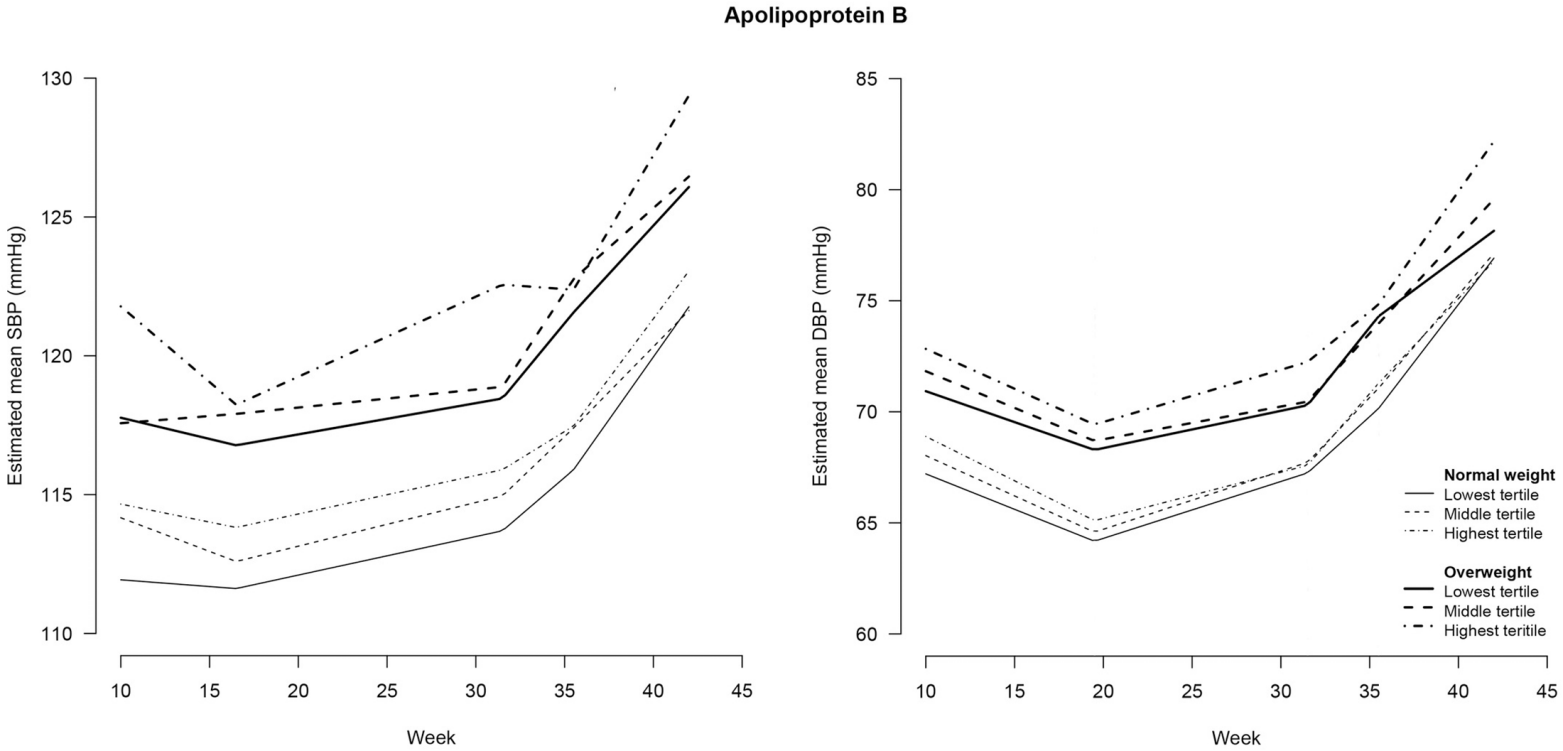
Blood pressure during pregnancy stratified for maternal weight status and tertiles of Apolipoprotein B^#^. SBP = systolic blood pressure; DBP = diastolic blood pressure. # Apolipoprotein B was determined in non-fasting blood samples drawn at a median of 13 (IQR = 12–14) weeks of gestation.

**Fig 7:**
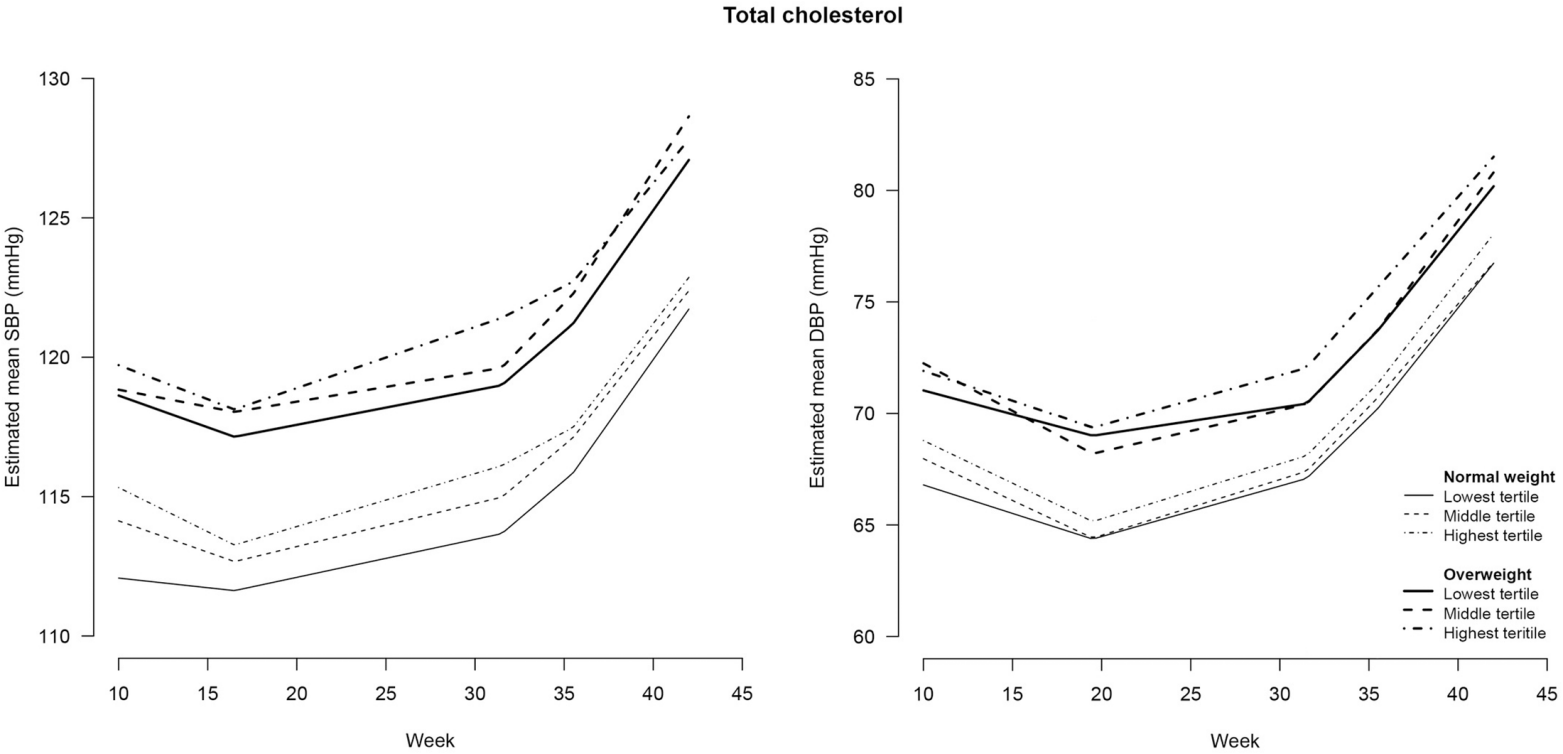
Blood pressure during pregnancy stratified for maternal weight status and tertiles of total cholesterol^#^. SBP = systolic blood pressure; DBP = diastolic blood pressure. # total cholesterol was determined in non-fasting blood samples drawn at a median of 13 (IQR = 12–14) weeks of gestation.

Figs 3–7 show the BP course for normal weight and overweight women stratified by lipid status. Although some differences can be seen in the associations between maternal pre-pregnancy weight status, early pregnancy lipid profile and BP course during pregnancy, adding the interaction terms between pre-pregnancy weight status, lipid status and time did not significantly improve the model. Thus, most maternal early pregnancy lipids did not modify the assocation between pre-pregnancy weight status and BP during pregnancy. However, in the SBP course the overall interaction with TG, and in the DBP course the overall interaction with FFA, were almost significant (p = 0.053 and p = 0.064, respectively). On further analysis, we found that overweight women in the highest tertile of TG had no decrease in SBP in the first period and a smaller increase in the second period of pregnancy (p<0.05) compared with the other women. Moreover, in the third time period, overweight women in the highest tertile of FFA had a steeper increase in DBP followed by a smaller increase in the last time period, compared with the other women.

### Sensitivity analyses

All analyses were repeated stratified by no complications (gestational age at birth ≥37 weeks and no gestational hypertensive disorders; S1 File) vs complications in pregnancy (gestational age at birth <37 weeks and/or PIH/PE; S2 File). In the subsample with no complication, SBP and DBP pattern were similar to the total study population, but differences between groups were similar. Adding an interaction term between time period, prepregnancy BMI and lipids did not improve the model fit for DBP, but for DBP the model fitted better with the interaction term was added to the models with TG, ApoA and FFA. For these lipids, differences between lipids were stable during the first 30 weeks of pregnancy, but hereafter, differences between the lipid tertiles diminished. The analyses in the subgroup with complications showed similar results with again smaller differences between women with overweight compared to women with normal weight. However, no clear patterns with maternal lipids could be determined.

## Discussion

This study shows that both pre-pregnancy weight status and early pregnancy lipid profile have an independent effect on BP course during pregnancy. Apart from a higher BP from the start, the BP course does not differ according to maternal weight status. Women with higher lipid levels in early pregnancy also have higher SBP and DBP from the start; however, if these women are also overweight, their DBP is increased even more. We found no evidence for our hypothesis that overweight women with a more atherogenic lipid profile have the most detrimental BP course.

Other studies investigating the association between pre-pregnancy weight status and BP course during pregnancy found (as confirmed by our study) that overweight women had both a higher SBP (range: 4.0–8.7 mmHg) and DBP (range: 1.6–5.6 mmHg) from the start of pregnancy onwards.[2, 5–7] Moreover, in our study and in a similar cohort these differences remained stable over time.[2] Two other studies reported that these differences decreased slightly over time, as normal weight women had a more rapid increase in BP at the end of pregnancy compared to overweight and obese women.[6, 7] To our knowledge, no other studies have examined the independent effect of maternal lipids, or the combined effect of maternal pBMI and lipids, on BP course during pregnancy. Other studies found a positive association between maternal lipids and the risk of hypertensive disorders in pregnancy, which suggests an influence of the lipid level on the BP course.[13–17] Our results add to this knowledge by showing that this increased risk might be caused by a higher BP at the very start of the pregnancy. Moreover, our DBP results suggest a smaller decrease in BP in early pregnancy and a stronger increase at the end of pregnancy in those women in the highest TG tertile. This pattern is associated with an increased risk for hypertensive disorders.[13–17]

### Strengths and limitations

The present investigation was based on a large prospective cohort study with (on average) 10 serial measurements of BP recordings throughout each individual pregnancy. The model we used took into account: i) the correlation of all BP measurements within each individual woman, as well as ii) the gestational age at birth, since women with hypertensive disorders of pregnancy give birth earlier than women with normotensive pregnancies. To examine the effect of pBMI and maternal lipids on BP course, we excluded women with both pre-existent and gestational diabetes, pre-existent hypertension and women using lipid-lowering medication. Moreover, we were able to filter out the BP measurements taken after medication was started for high BP. This resulted in a more reliable estimate of the BP course based on maternal pBMI and lipids, and not influenced by medical interventions. On the other hand, as women with gestational diabetes have higher BPs during pregnancy,[24] and women who used hypertensive medication in our study were more often overweight, excluding them most likely resulted in an underestimation of the effects of maternal weight status on BP course during pregnancy. Most other studies also excluded women with pre-existent/gestational hypertension and/or diabetes.[2, 5, 6] Furthermore, results of a sensitivity analysis in a subsample without complications yielded similar results as in the whole study sample, however, in a sensitivity analysis a group with complications no associations with maternal lipids were found. This could be the result of other unknown underlying mechanisms causing hypertensive disorders and/or preterm birth. Because the present study had a large study population, we were able to correct for a wide range of covariates. Nevertheless, we did not measure some important covariates such as, for instance, nutritional status/dietary patterns and gestational weight gain;[5] thus, potentially, residual confounding might overestimate our results. Our non-response analysis also showed that the women included in this study were slightly healthier compared to the sample that lacked BP and/or lipid measurements; this could also have led to an underestimation of the true effect. Moreover, also the use of self-reported data could have led to an underestimation of the true effect as women tend to overestimate their height and underestimate their weight.[25] This could have resulted in an underestimation of BMI and misclassification of overweight women in the normal weight group, diminishing the differences found between the normal weight and overweight groups.

Finally, maternal lipids were only measured once, non-fasting in early pregnancy, and randomly during the day. Since maternal lipids fluctuate during pregnancy, more extreme increases in the second half of pregnancy could, for instance, be more directly related to BP increases during this second half of pregnancy. However, other studies found that increased maternal lipids in early pregnancy were already associated with an increased risk of developing hypertensive disorders later in pregnancy.[14, 16] Therefore, we expect that it is possible to relate early pregnancy lipids to BP course during pregnancy; however, our evidence would have been strengthened by multiple lipid measurements in pregnancy. Also, the non-fasting state could have diluted our results for TG and TC as normal food intake is known to alter TG and TC, but not ApoA1 and ApoB.[26] Furthermore, it has been shown that non-fasting levels of TG in women predicted cardiovascular events more strongly than fasting levels.[27] Therefore, using non-fasting lipids could be more predictive of BP course than fasting lipids.

### Underlying mechanisms

Obese pregnant women have increased lipids,[10–12] and a dysregulated vascular and immune system.[28] All three factors can contribute to the development of hypertension or pre-eclampsia. Obesity affects endothelial dysfunction,[28] whereas lipids have a direct and also an indirect effect on endothelial dysfunction.[18] Proper endothelial function is important as the endothelial produces (amongst others) nitric oxide (NO), a vasodilator which decreases BP. In non-pregnant women, ApoA1 was found to be positively correlated with elastin peptides,[29] which induce NO-dependent vasodilatation.[30] On the other hand, although TG are not atherogenic by itself, high levels of non-fasting TG are an indicator of cytotoxic remnants of ApoB-lipoproteins like chylomicron and very low density lipoproteins.[31] Moreover, TG-rich ApoB-lipoproteins can penetrate the arterial intima, promoting endothelial damage.[32] FFA plays an important role here, as they promote low-density lipoprotein and cholesterol-rich remnant particles to enter the intima.[18] Moreover, FFA can increase oxidative stress,[18] causing oxidation of ApoB-lipoproteins like LDL, which decreases vasodilatation through decreased NO synthesis and release and degradation of NO.[33]

Furthermore, also a dysregulated immune system can contribute to an increased BP. Inflammatory factors are increased to a greater extent in overweight/obese women compared to normal weight women.[28, 34] These inflammatory markers (TNF-α and interleukin-6) can be secreted from adipocytes and stimulate the liver to produce C-reactive protein (CRP).[35] Our study shows that lipids do not mediate the association between obesity and BP course during pregnancy. CRP was reported to be positively associated with BP in pregnancy, but correcting for pBMI attenuated these associations.[36, 37] This suggests a mediating role for the inflammatory markers, like CRP, in the association between pBMI and BP course during pregnancy, apart from the independent role of obesity and lipids on BP.

### Conclusion

This study shows that overweight/obese women in the highest tertile of maternal lipids start their pregnancy with a 7 mmHg higher SBP and a 4.9 mmHg higher DBP. Both maternal pBMI and maternal lipids have an independent effect on BP during pregnancy. However, BP course was not changed by maternal pBMI or maternal lipids. Improving maternal pBMI and lipid profile in early pregnancy might improve BP during pregnancy.

## Supporting information

S1 Table Analysis of the response and non-response groups. * Non-response group consisted of women who participated in the ABCD-study, but did not give their consent for the biomarker study (no information on early pregnancy lipids) or with less than 3 blood pressure measurements or with first blood pressure measured after 20 weeks of gestation. Exclusion criteria applied to the response group were also applied to the non-response group.
(DOC)

S1 File Sensitivity analyses “no complications”. All analyses were repeated on this subsample of women with no complications during pregnancy (gestational age at birth ≥37 weeks and no PIH/PE).
(DOC)

S2 File Sensitivity analyses “complications”. All analyses were repeated on this subsample of women with complications during pregnancy (gestational age at birth <37 weeks and/or PIH/PE).
(DOC)

## References

[1] O'Brien TE, Ray JG, Chan WS. Maternal body mass index and the risk of preeclampsia: a systematic overview. Epidemiology. 2003;14(3):368–74. 12859040 10.1097/00001648-200305000-00020

[2] Gaillard R, Steegers EA, Hofman A, Jaddoe VW. Associations of maternal obesity with blood pressure and the risks of gestational hypertensive disorders. The Generation R Study. J Hypertens. 2011;29(5):937–44. 10.1097/HJH.0b013e328345500c 21430559

[3] Khan KS, Wojdyla D, Say L, Gulmezoglu AM, Van Look PF. WHO analysis of causes of maternal death: a systematic review. Lancet. 2006;367(9516):1066–74. 10.1016/S0140-6736(06)68397-9 16581405

[4] Hutcheon JA, Lisonkova S, Joseph KS. Epidemiology of pre-eclampsia and the other hypertensive disorders of pregnancy. Best Pract Res Clin Obstet Gynaecol. 2011;25(4):391–403. 10.1016/j.bpobgyn.2011.01.006 21333604

[5] Magriples U, Boynton MH, Kershaw TS, Duffany KO, Rising SS, Ickovics JR. Blood pressure changes during pregnancy: impact of race, body mass index, and weight gain. Am J Perinatol. 2013;30(5):415–24. 10.1055/s-0032-1326987 23059493 PMC3938313

[6] Miller RS, Thompson ML, Williams MA. Trimester-specific blood pressure levels in relation to maternal pre-pregnancy body mass index. Paediatr Perinat Epidemiol. 2007;21(6):487–94. 10.1111/j.1365-3016.2007.00871.x 17937733

[7] Macdonald-Wallis C, Tilling K, Fraser A, Nelson SM, Lawlor DA. Established preeclampsia risk factors are related to patterns of blood pressure change in normal term pregnancy: findings from the Avon Longitudinal Study of Parents and Children. J Hypertens. 2011;29(9):1703–11. 10.1097/HJH.0b013e328349eec6 21841545

[8] Torgersen KL, Curran CA. A systematic approach to the physiologic adaptations of pregnancy. Crit Care Nurs Q. 2006;29(1):2–19. 16456359 10.1097/00002727-200601000-00002

[9] Macdonald-Wallis C, Lawlor DA, Fraser A, May M, Nelson SM, Tilling K. Blood pressure change in normotensive, gestational hypertensive, preeclamptic, and essential hypertensive pregnancies. Hypertension. 2012;59(6):1241–8. 10.1161/HYPERTENSIONAHA.111.187039 22526257 PMC3378662

[10] Gademan MG, Vermeulen M, Oostvogels AJ, Roseboom TJ, Visscher TL, van Eijsden M, et al Maternal prepregancy BMI and lipid profile during early pregnancy are independently associated with offspring's body composition at age 5–6 years: the ABCD study. PLoS One. 2014;9(4):e94594 10.1371/journal.pone.0094594 24740157 PMC3989215

[11] Scifres CM, Catov JM, Simhan HN. The impact of maternal obesity and gestational weight gain on early and mid-pregnancy lipid profiles. Obesity (Silver Spring). 2014;22(3):932–8. 23853155 10.1002/oby.20576 PMC4362720

[12] Farias DR, Franco-Sena AB, Vilela A, Lepsch J, Mendes RH, Kac G. Lipid changes throughout pregnancy according to pre-pregnancy BMI: results from a prospective cohort. BJOG. 2016;123(4):570–8. 10.1111/1471-0528.13293 25639335

[13] Vrijkotte TG, Krukziener N, Hutten BA, Vollebregt KC, van Eijsden M, Twickler MB. Maternal lipid profile during early pregnancy and pregnancy complications and outcomes: the ABCD study. J Clin Endocrinol Metab. 2012;97(11):3917–25. 10.1210/jc.2012-1295 22933545

[14] Enquobahrie DA, Williams MA, Butler CL, Frederick IO, Miller RS, Luthy DA. Maternal plasma lipid concentrations in early pregnancy and risk of preeclampsia. Am J Hypertens. 2004;17(7):574–81. 10.1016/j.amjhyper.2004.03.666 15233976

[15] Clausen T, Djurovic S, Henriksen T. Dyslipidemia in early second trimester is mainly a feature of women with early onset pre-eclampsia. BJOG. 2001;108(10):1081–7. 11702841 10.1111/j.1471-0528.2001.00247.x

[16] Kandimalla BH, Sirjusingh A, Nayak BS, Maiya SS. Early antenatal serum lipid levels and the risk of pre-eclampsia in Trinidad and Tobago. Arch Physiol Biochem. 2011;117(4):215–21. 10.3109/13813455.2010.543137 21226541

[17] Ray JG, Diamond P, Singh G, Bell CM. Brief overview of maternal triglycerides as a risk factor for pre-eclampsia. BJOG. 2006;113(4):379–86. 10.1111/j.1471-0528.2006.00889.x 16553649

[18] Sattar N, Petrie JR, Jaap AJ. The atherogenic lipoprotein phenotype and vascular endothelial dysfunction. Atherosclerosis. 1998;138(2):229–35. 9690905 10.1016/s0021-9150(98)00037-9

[19] Gratacos E. Lipid-mediated endothelial dysfunction: a common factor to preeclampsia and chronic vascular disease. Eur J Obstet Gynecol Reprod Biol. 2000;92(1):63–6. 10986436 10.1016/s0301-2115(00)00427-9

[20] Savvidou MD, Hingorani AD, Tsikas D, Frolich JC, Vallance P, Nicolaides KH. Endothelial dysfunction and raised plasma concentrations of asymmetric dimethylarginine in pregnant women who subsequently develop pre-eclampsia. Lancet. 2003;361(9368):1511–7. 10.1016/S0140-6736(03)13177-7 12737861

[21] van Eijsden M, Vrijkotte TG, Gemke RJ, van der Wal MF. Cohort profile: the Amsterdam Born Children and their Development (ABCD) study. Int J Epidemiol. 2011;40(5):1176–86. 10.1093/ije/dyq128 20813863

[22] WHO. Global Database on Body Mass Index 2014 [cited 2014 March 10th]. www.who.int/bmi.

[23] Buuren van S G-O K. MICE: Multivariate Imputation by Chained Equations in R. 2010;10:1–68.

[24] Carpenter MW. Gestational diabetes, pregnancy hypertension, and late vascular disease. Diabetes Care. 2007;30 Suppl 2:S246–S50. 17596480 10.2337/dc07-s224

[25] Stewart AW, Jackson RT, Ford MA, Beaglehole R. Underestimation of relative weight by use of self-reported height and weight. AmJEpidemiol. 1987;125(1):122–6. 10.1093/oxfordjournals.aje.a114494 3788941

[26] Langsted A, Freiberg JJ, Nordestgaard BG. Fasting and nonfasting lipid levels: influence of normal food intake on lipids, lipoproteins, apolipoproteins, and cardiovascular risk prediction. Circulation. 2008;118(20):2047–56. 10.1161/CIRCULATIONAHA.108.804146 18955664

[27] Bansal S, Buring JE, Rifai N, Mora S, Sacks FM, Ridker PM. Fasting compared with nonfasting triglycerides and risk of cardiovascular events in women. JAMA. 2007;298(3):309–16. 10.1001/jama.298.3.309 17635891

[28] Ramsay JE, Ferrell WR, Crawford L, Wallace AM, Greer IA, Sattar N. Maternal obesity is associated with dysregulation of metabolic, vascular, and inflammatory pathways. J Clin Endocrinol Metab. 2002;87(9):4231–7. 10.1210/jc.2002-020311 12213876

[29] Bizbiz L, Alperovitch A, Robert L. Aging of the vascular wall: serum concentration of elastin peptides and elastase inhibitors in relation to cardiovascular risk factors. The EVA study. Atherosclerosis. 1997;131(1):73–8. 9180247 10.1016/s0021-9150(97)06076-0

[30] Faury G, Ristori MT, Verdetti J, Jacob MP, Robert L. Effect of elastin peptides on vascular tone. J Vasc Res. 1995;32(2):112–9. 7734657 10.1159/000159084

[31] Zilversmit DB. Atherogenesis: a postprandial phenomenon. Circulation. 1979;60(3):473–85. 222498 10.1161/01.cir.60.3.473

[32] Nordestgaard BG, Wootton R, Lewis B. Selective retention of VLDL, IDL, and LDL in the arterial intima of genetically hyperlipidemic rabbits in vivo. Molecular size as a determinant of fractional loss from the intima-inner media. Arterioscler Thromb Vasc Biol. 1995;15(4):534–42. 7749867 10.1161/01.atv.15.4.534

[33] Chin JH, Azhar S, Hoffman BB. Inactivation of endothelial derived relaxing factor by oxidized lipoproteins. J Clin Invest. 1992;89(1):10–8. 10.1172/JCI115549 1309534 PMC442813

[34] Visser M, Bouter LM, McQuillan GM, Wener MH, Harris TB. Elevated C-reactive protein levels in overweight and obese adults. JAMA. 1999;282(22):2131–5. 10591334 10.1001/jama.282.22.2131

[35] Mohamed-Ali V, Pinkney JH, Coppack SW. Adipose tissue as an endocrine and paracrine organ. Int J Obes Relat Metab Disord. 1998;22(12):1145–58. 9877249 10.1038/sj.ijo.0800770

[36] de Jonge LL, Steegers EA, Ernst GD, Lindemans J, Russcher H, Hofman A, et al C-reactive protein levels, blood pressure and the risks of gestational hypertensive complications: the Generation R Study. J Hypertens. 2011;29(12):2413–21. 10.1097/HJH.0b013e32834c58e5 22002335

[37] Wolf M, Kettyle E, Sandler L, Ecker JL, Roberts J, Thadhani R. Obesity and preeclampsia: the potential role of inflammation. Obstet Gynecol. 2001;98(5 Pt 1):757–62. 11704165 10.1016/s0029-7844(01)01551-4

