# Supplementary material for: Pre-pregnancy weight status, early pregnancy lipid profile and blood pressure course during pregnancy: The ABCD study
Source: PLoS One. 2017 May 19;12(5):e0177554. doi: 10.1371/journal.pone.0177554 (PMC5438136; doi:10.1371/journal.pone.0177554)
Supplement: S1 Table — * Non-response group consisted of women who participated in the ABCD-study, but did not give their consent for the biomarker study (no information on early pregnancy lipids) or with less than 3 blood pressure measurements or with first blood pressure measured after 20 weeks of gestation. Exclusion criteria applied to the response group were also applied to the non-response group. (DOC) [file pone.0177554.s001.doc]

**S1 Table. Analysis of the response and non-response groups.**

|  |  | | **Response**  **n=3100** | **Non-Response^*^**  **n=4169** | | | |  | | |  |
| --- | --- | --- | --- | --- | --- | --- | --- | --- | --- | --- | --- |
|  | | | *%/mean (SD)* | | *%/mean (SD)* | | *p-value* | | |  | |
| *Maternal characteristics* | | |  |  | | | |  | | |  |
| Age (years) | |  | 31.1 (4.7) | 30.3 (5.6) | | | | p<0.001 | | |  |
| Body mass index (kg/m**^2^**) | | | 22.7 (3.6) | 23.2 (4.2) | | | | p<0.001 | | |  |
| Education (years) | |  | 9.5 (3.7) | 8.1 (4.3) | | | | p<0.001 | | |  |
| Ethnicity (%) | | Dutch | 63 | 45 | | | |  | | |  |
|  | | Surinamese-Hindu | 1 | 2 | | | |  | | |  |
|  | | Black Caribbean | 4 | 6 | | | |  | | |  |
|  | | Turkish | 4 | 6 | | | | p<0.001 | | |  |
|  | | Moroccan | 6 | 12 | | | |  | | |  |
|  | | Ghanaian | 1 | 3 | | | |  | | |  |
|  | | Other | 21 | 26 | | | |  | | |  |
| Parity (%) | | Nulliparous | 58 | 54 | | | | p<0.001 | | |  |
|  | | Multiparous | 42 | 46 | | | |  | | |  |
| Smoking | | No | 91 | 91 | | | | p=1.000 | | |  |
| during pregnancy (%) | | Yes | 9 | 9 | | | |  | | |  |
| Alcohol consumption | | No | 74 | 83 | | | | p<0.001 | | |  |
| during pregnancy (%) | | Yes | 26 | 17 | | | |  | | |  |
|  | | |  |  | | | |  | | |  |
| *Offspring’s characteristics* | | |  |  | | | |  | | |  |
| Foetal sex (%) | | Male | 49 | | | 52 | | | p=0.039 | | |
|  | | Female | 51 | | | 48 | | |  | | |
| Gestational age at birth (days) | | Mean (SD) | 279 (12.9) | | | 278 (13.3) | | | p=0.012 | | |

* Non-response group consisted of women who participated in the ABCD-study, but did not give their consent for the biomarker study (no information on early pregnancy lipids) or with less than 3 blood pressure measurements or with first blood pressure measured after 20 weeks of gestation. Exclusion criteria applied to the response group were also applied to the non-response group.
