# Supplementary material for: Pre-pregnancy weight status, early pregnancy lipid profile and blood pressure course during pregnancy: The ABCD study
Source: PLoS One. 2017 May 19;12(5):e0177554. doi: 10.1371/journal.pone.0177554 (PMC5438136; doi:10.1371/journal.pone.0177554)
Supplement: S1 File — All analyses were repeated on this subsample of women with no complications during pregnancy (gestational age at birth ≥37 weeks and no PIH/PE). (DOC) [file pone.0177554.s002.doc]

**Table 1. Characteristics of the subgroup without complications^#^ (N=2502)**

|  | **Normal weight**  **(n=2082)** | | **Overweight (n=420)** |  | |
| --- | --- | --- | --- | --- | --- |
|  | *%/mean (SD)* | *%/mean (SD)* | | | *p-value* |
| Age (years) | 31.3 (4.6) | 31.0 (5.1) | | | P=0.313 |
| Body mass index (kg/m2) | 21.4 (1.8) | 28.0 (3.3) | | | P<0.001 |
| Education (years) | 9.8 (3.5) | 7.8 (4.1) | | | P<0.001 |
| Ethnicity |  |  | | | P<0.001 |
| - Dutch | 64.5 | 46.7 | | |  |
| - Surinamese-Hindu | 1.5 | 1.4 | | |  |
| - Black Caribbean | 3.2 | 8.8 | | |  |
| - Turkish | 3.7 | 6.4 | | |  |
| - Moroccan | 3.7 | 16.2 | | |  |
| - Ghanaian | 0.4 | 3.3 | | |  |
| - Other | 23.0 | 17.1 | | |  |
| % Nulliparous | 57.9 | 41.2 | | | P<0.001 |
| % Smoking during pregnancy | 9.6 | 9.5 | | | P=1.000 |
| % Alcohol consumption during pregnancy | 30.2 | 15.5 | | | P<0.001 |
| Gestational age at lipid measurement (days) | 92 (1) | 94 (17) | | | P=0.143 |
| Triglycerides (mmol/L)* | 1.53 (0.58) | 1.69 (0.60) | | | P<0.001 |
| Free fatty acids (mmol/L)* | 0.31 (0.16) | 0.37 (0.19) | | | P<0.001 |
| Total cholesterol (mmol/L)* | 5.37 (0.99) | 5.51 (1.03) | | | P=0.013 |
| Apolipoprotein A1 (g/L)* | 1.65 (0.24) | 1.61 (0.24) | | | P=0.007 |
| Apolipoprotein B (g/L)* | 0.81 (0.20) | 0.88 (0.21) | | | P<0.001 |
| % Males | 48.9 | 49.3 | | | P=0.915 |
| Gestational age at birth (days) | 281 (8) | 281 (9) | | | P=0.515 |

* lipids were interpolated to 10 weeks of gestation
# Women who gave birth after 37 weeks of gestation and did not have pregnancy induced hypertension or preeclampsia

**Table 2. Lipid tertiles^#^ of the subgroup without complications**

|  |  | **Lowest tertile** | | **Middle tertile** | | **Highest tertile** | |
| --- | --- | --- | --- | --- | --- | --- | --- |
|  | **N_total_** | **N** | **%** | **N** | **%** | **N** | **%** |
| **TG** | 2449 | 823 | 33.6 | 834 | 34.1 | 792 | 32.3 |
| **TC** | 2450 | 843 | 34.4 | 806 | 32.9 | 801 | 32.7 |
| **ApoA_1_** | 1759 | 613 | 34.8 | 582 | 33.1 | 564 | 32.1 |
| **ApoB** | 1772 | 609 | 34.4 | 589 | 33.2 | 574 | 32.4 |
| **FFA** | 1681 | 573 | 34.1 | 561 | 33.4 | 547 | 32.5 |

# tertiles were kept equal to tertiles in the total study population

**Table 3. Association between pre-pregnancy BMI and systolic blood pressure course during pregnancy, with maternal lipids**

|  |  | **Mean difference in average systolic blood pressure (mmHg)** | | | | |
| --- | --- | --- | --- | --- | --- | --- |
|  |  | **pBMI** | | **Lipids#** | | |
|  |  | Normal weight | Overweight | Lowest tertile | Middle tertile | Highest tertile |
| **Model 1** |  | Ref | 2.5 (1.8-3.2) | - | - | - |
| **Model 2** |  | Ref | 3.4 (2.7-4.1) | - | - | - |
| **Model 3** | + TG | Ref | 3.3 (2.6-4.1) | Ref | 0.8 (0.2-1.4) | 1.4 (0.7-2.0) |
|  | + TC | Ref | 3.4 (2.6-4.1) | Ref | 0.8 (0.1-1.4) | 1.3 (0.7-1.9) |
|  | + ApoA1 | Ref | 3.4 (2.5-4.2) | Ref | 0.6 (-0.1-1.3) | 0.7 (-0.0-1.5) |
|  | + ApoB | Ref | 3.2 (2.4-4.0) | Ref | 0.9 (0.1-1.6) | 1.8 (1.0-2.5) |
|  | + FFA | Ref | 3.3 (2.4-4.2) | Ref | 0.4 (-0.3-1.2) | 1.1 (0.3-1.8) |

Model 1 – crude model; model 2 –adjusted for: maternal age, education, ethnicity, parity, smoking and alcohol use during pregnancy and sex of the foetus; model 3 –adjusted for model 2 + individual lipids separately.

TG = triglycerides (mmol/L); TC = total cholesterol (mmol/L); ApoA1 = Apolipoprotein A1 (mg/dL); ApoB = Apolipoprotein B (mg/dL); FFA = Free Fatty Acids (mmol/L) # lipids were determined in non-fasting blood samples drawn at a median of 13 (IQR=12-14 ) weeks of gestation.

**Table 4. Association between pre-pregnancy body mass index (pBMI) and diastolic blood pressure course during pregnancy, with maternal lipids**

|  |  | **Mean difference in average diastolic blood pressure (mmHg)** | | | | |
| --- | --- | --- | --- | --- | --- | --- |
|  |  | **pBMI** | | **Lipids#** | | |
|  |  | Normal weight | Overweight | Lowest tertile | Middle tertile | Highest tertile |
| **Model 1** |  | Ref | 2.0 (1.5-2.6) | - | - | - |
| **Model 2** |  | Ref | 2.7 (2.1-3.3) | - | - | - |
| **Model 3** | + TG | Ref | 2.7 (2.1-3.2) | Ref | * | 0.8 (0.2-1.3) |
|  | + TC | Ref | 2.7 (2.1-3.3) | Ref | 0.0 (-0.5-0.5) | 0.6 (0.1-1.1) |
|  | + ApoA1 | Ref | 2.6 (1.9-3.2) | Ref | -0.3 (-0.9-0.3) | 0.3 (-0.3-0.9) |
|  | + ApoB | Ref | 2.5 (1.8-3.2) | Ref | 0.2 (-0.4-0.8) | 0.7 (0.1-1.3) |
|  | + FFA | Ref | 2.5 (1.9-3.2) | Ref | 0.4 (-0.2-1.0) | 0.7 (0.1-1.3) |

Model 1 – crude model; model 2 –adjusted for: maternal age, education, ethnicity, parity, smoking and alcohol use during pregnancy and sex of the foetus; model 3 – adjusted for model 2 + individual lipids separately.
TG = triglycerides (mmol/L); TC = total cholesterol (mmol/L); ApoA1 = Apolipoprotein A1 (mg/dL); ApoB = Apolipoprotein B (mg/dL); FFA = Free Fatty Acids (mmol/L) # lipids were determined in non-fasting blood samples drawn at a median of 13 (IQR=12-14 ) weeks of gestation.
* Significant interactions between tertiles of lipids and time period; blood pressure course for these tertiles are described in detail in the text

**Figure 3. Blood pressure during pregnancy stratified for maternal weight status and tertiles of triglycerides#.**

SBP= systolic blood pressure; DBP= diastolic blood pressure

# triglycerides were determined in non-fasting blood samples drawn at a median of 13 (IQR=12-14 ) weeks of gestation.

**Figure 4. Blood pressure during pregnancy stratified for maternal weight status and tertiles of total cholesterol#.**

SBP= systolic blood pressure; DBP= diastolic blood pressure

# total cholesterol was determined in non-fasting blood samples drawn at a median of 13 (IQR=12-14 ) weeks of gestation.

**Figure 5. Blood pressure during pregnancy stratified for maternal weight status and tertiles of Apolipoprotein A1#.**

SBP= systolic blood pressure; DBP= diastolic blood pressure

# Apolipoprotein A1 was determined in non-fasting blood samples drawn at a median of 13 (IQR=12-14 ) weeks of gestation.

**Figure 6. Blood pressure during pregnancy stratified for maternal weight status and tertiles of Apolipoprotein B#.**

SBP= systolic blood pressure; DBP= diastolic blood pressure

# Apolipoprotein B was determined in non-fasting blood samples drawn at a median of 13 (IQR=12-14 ) weeks of gestation.

**Figure 7. Blood pressure during pregnancy stratified for maternal weight status and tertiles of free fatty acids#.**

SBP= systolic blood pressure; DBP= diastolic blood pressure

# free fatty acids were determined in non-fasting blood samples drawn at a median of 13 (IQR=12-14 ) weeks of gestation.
