# Supplementary material for: Pre-pregnancy weight status, early pregnancy lipid profile and blood pressure course during pregnancy: The ABCD study
Source: PLoS One. 2017 May 19;12(5):e0177554. doi: 10.1371/journal.pone.0177554 (PMC5438136; doi:10.1371/journal.pone.0177554)
Supplement: S2 File — All analyses were repeated on this subsample of women with complications during pregnancy (gestational age at birth <37 weeks and/or PIH/PE). (DOC) [file pone.0177554.s003.doc]

**Table 1. Characteristics of the subgroup with complications^#^ (N=598)**

|  | **Normal weight**  **(n=418)** | | **Overweight (n=180)** |  | |
| --- | --- | --- | --- | --- | --- |
|  | *%/mean (SD)* | *%/mean (SD)* | | | *p-value* |
| Age (years) | 30.5 (4.8) | 31.2 (4.3) | | | P=0.106 |
| Body mass index (kg/m2) | 21.7 (1.9) | 28.9 (3.6) | | | P<0.001 |
| Education (years) | 9.6 (3.5) | 8.6 (4.0) | | | P=0.003 |
| Ethnicity |  |  | | | P<0.001 |
| - Dutch | 69.4 | 58.9 | | |  |
| - Surinamese-Hindu | 1.2 | 1.1 | | |  |
| - Black Caribbean | 2.9 | 10.0 | | |  |
| - Turkish | 2.9 | 3.9 | | |  |
| - Moroccan | 3.8 | 5.6 | | |  |
| - Ghanaian | 0.2 | 3.9 | | |  |
| - Other | 19.6 | 16.7 | | |  |
| % Nulliparous | 74.2 | 62.2 | | | P<0.004 |
| % Smoking during pregnancy | 9.8 | 5.6 | | | P=0.110 |
| % Alcohol consumption during pregnancy | 23.2 | 15.0 | | | P=0.028 |
| Gestational age at lipid measurement (days) | 91 (15) | 92 (16) | | | P=0.529 |
| Triglycerides (mmol/L)* | 1.57 (0.62) | 1.80 (0.72) | | | P<0.001 |
| Free fatty acids (mmol/L)* | 0.31 (0.14) | 0.39 (0.23) | | | P=0.001 |
| Total cholesterol (mmol/L)* | 5.43 (0.93) | 5.59 (0.98) | | | P=0.056 |
| Apolipoprotein A1 (g/L)* | 1.69 (0.26) | 1.68 (0.23) | | | P=0.915 |
| Apolipoprotein B (g/L)* | 0.82 (0.20) | 0.88 (0.19) | | | P=0.001 |
| % Males | 50.2 | 47.8 | | | P=0.594 |
| Gestational age at birth (days) | 270 (22) | 274 (21) | | | P=0.035 |
| % Preterm | 27.5 | 20.0 | | | P=0.064 |

* lipids were interpolated to 10 weeks of gestation
# women who gave birth before 37 weeks of pregnancy or who developed pregnancy induced hypertension or preeclampsia

**Table 2. Lipid tertiles^#^ of the subgroup with complications**

|  |  | **Lowest tertile** | | **Middle tertile** | | **Highest tertile** | |
| --- | --- | --- | --- | --- | --- | --- | --- |
|  | **N_total_** | **N** | **%** | **N** | **%** | **N** | **%** |
| **TG** | 586 | 189 | 32.3 | 178 | 30.4 | 219 | 37.4 |
| **TC** | 586 | 168 | 28.7 | 207 | 35.3 | 211 | 36.0 |
| **ApoA_1_** | 417 | 113 | 27.1 | 144 | 34.5 | 160 | 38.4 |
| **ApoB** | 421 | 121 | 28.7 | 143 | 34.0 | 157 | 37.3 |
| **FFA** | 398 | 120 | 30.2 | 132 | 33.2 | 146 | 36.7 |

# tertiles were kept equal to tertiles in the total study population

**Table 3 + Table 4**

We cannot display tables for the adverse outcome subset as we found interactions for both differences over time with prepregnancy BMI and also three-way-interactions between time, prepregnancy BMI and maternal lipids.

**Figure 2. Blood pressure course during pregnancy stratified for women with normal weight and overweight.**

1. *Systolic blood pressure (SBP): 10-16.5 weeks: estimated β=-0.2 mmHg/week (95% CI: -0.3;-0.1), 16.5-31.5 weeks: estimated β=0.2 mmHg/week (95% CI: 0.1;0.2), 31.5-35.5 weeks: estimated β=0.5 mmHg/week (95% CI: 0.4;0.6), 35.5-42 weeks: estimated β=0.9 mmHg/week (95% CI: 0.7;1.0)*
2. *Diastolic blood pressure (DBP): 10-19.5 weeks: estimated β=-0.3 mmHg/week (95% CI: -0.4;-0.3), 19.5-31.5 weeks: estimated β=0.2 mmHg/week (95% CI: 0.2;0.3), 31.5-35.5 weeks: estimated β=0.8 mmHg/week (95% CI: 0.7;0.9), 35.5-42 weeks: estimated β=1.0 mmHg/week (95% CI: 0.9;1.1)*

**Figure 3. Blood pressure during pregnancy stratified for maternal weight status and tertiles of triglycerides^#^.**

SBP= systolic blood pressure; DBP= diastolic blood pressure

# triglycerides were determined in non-fasting blood samples drawn at a median of 13 (IQR=12-14 ) weeks of gestation.

**Figure 4. Blood pressure during pregnancy stratified for maternal weight status and tertiles of total cholesterol^#^.**

SBP= systolic blood pressure; DBP= diastolic blood pressure

# total cholesterol was determined in non-fasting blood samples drawn at a median of 13 (IQR=12-14 ) weeks of gestation.

**Figure 5. Blood pressure during pregnancy stratified for maternal weight status and tertiles of Apolipoprotein A_1_^#^.**

SBP= systolic blood pressure; DBP= diastolic blood pressure

# Apolipoprotein A_1_ was determined in non-fasting blood samples drawn at a median of 13 (IQR=12-14 ) weeks of gestation.

**Figure 6. Blood pressure during pregnancy stratified for maternal weight status and tertiles of Apolipoprotein B^#^.**

SBP= systolic blood pressure; DBP= diastolic blood pressure

# Apolipoprotein B was determined in non-fasting blood samples drawn at a median of 13 (IQR=12-14 ) weeks of gestation.

**Figure 7. Blood pressure during pregnancy stratified for maternal weight status and tertiles of free fatty acids^#^.**

SBP= systolic blood pressure; DBP= diastolic blood pressure

# free fatty acids were determined in non-fasting blood samples drawn at a median of 13 (IQR=12-14 ) weeks of gestation.
